# A novel CpG ODN compound adjuvant enhances immune response to spike subunit vaccines of porcine epidemic diarrhea virus

**DOI:** 10.3389/fimmu.2024.1336239

**Published:** 2024-01-23

**Authors:** Yating Wang, Shijia Liu, Boshuo Li, Xinyao Sun, Qi Pan, Yuxin Zheng, Jia Liu, Yongqiang Zhao, Jingyu Wang, Liming Liu, Enqi Du

**Affiliations:** ^1^ College of Veterinary Medicine, Northwest A&F University, Yangling, China; ^2^ Nanjing JSIAMA Biopharmaceuticals Ltd., Nanjing, China; ^3^ Yangling Carey Biotechnology Co., Ltd., Yangling, China

**Keywords:** PEDV, CpG ODN, subunit vaccine, compound adjuvant, immune response

## Abstract

CpG oligodeoxynucleotides (CpG ODNs) boost the humoral and cellular immune responses to antigens through interaction with Toll-like receptor 9 (TLR9). These CpG ODNs have been extensively utilized in human vaccines. In our study, we evaluated five B-type CpG ODNs that have stimulatory effects on pigs by measuring the proliferation of porcine peripheral blood mononuclear cells (PBMCs) and assessing interferon gamma (IFN-γ) secretion. Furthermore, this study examined the immunoenhancing effects of the MF59 and CpG ODNs compound adjuvant in mouse and piglet models of porcine epidemic diarrhea virus (PEDV) subunit vaccine administration. The *in vitro* screening revealed that the CpG ODN named CpG5 significantly stimulated the proliferation of porcine PBMCs and elevated IFN-γ secretion levels. In the mouse vaccination model, CpG5 compound adjuvant significantly bolstered the humoral and cellular immune responses to the PEDV subunit vaccines, leading to Th1 immune responses characterized by increased IFN-γ and IgG2a levels. In piglets, the neutralizing antibody titer was significantly enhanced with CpG5 compound adjuvant, alongside a considerable increase in CD8+ T lymphocytes proportion. The combination of MF59 adjuvant and CpG5 exhibits a synergistic effect, resulting in an earlier, more intense, and long-lasting immune response in subunit vaccines for PEDV. This combination holds significant promise as a robust candidate for the development of vaccine adjuvant.

## 1 Introduction

Porcine epidemic diarrhea virus (PEDV) belongs to the genus *Alphacoronavirus* of the coronavirus family and can cause acute diarrhea, vomiting, dehydration and death in newborn piglets. PEDV first appeared in the United Kingdom in the late 1970s (1), and the PEDV strain CV777 was first isolated in Belgium (2). Since 2010, outbreaks of highly virulent PEDV strains have occurred in China (3), followed by the United States and Mexico (4, 5), Japan (6), South Korea (7), and Vietnam (8). Other countries have subsequently reported outbreaks, leading to significant economic losses in the global swine industry.

Vaccination remains the primary method for preventing and controlling diarrhea in piglets. Currently, inactivated and live attenuated vaccines are predominantly used for prevention and control in China. However, due to the continuous variation of the strain, existing porcine epidemic diarrhea vaccines are insufficient to control the prevalence of variants (9). In recent years, the application of genetic engineering technology to develop novel subunit vaccines has gained prominence, with notable examples including vaccines for COVID-19 (10–12), influenza (13, 14), respiratory syncytial virus (15), and Ebola (16). Similar to other coronaviruses, the PEDV spike glycoprotein (S) plays an essential role in cell attachment and virus-host membrane fusion, facilitating the entry of the virus into host cells (17, 18). The S protein is present on the surface of the coronavirus in two conformations: prefusion and postfusion (19, 20). Previous studies have demonstrated that maintaining the prefusion conformation of the coronavirus S protein can enhance its ability to trigger an immune response (21, 22). In this study, two amino acids in the S2 subunit of the wild-type PEDV S protein were mutated based on protein structure analysis. This mutation aimed to enhance the stability of the S protein conformation, promoting a predominantly trimeric prefusion state. The S protein is crucial for eliciting antibody responses in the host and is a promising immunogen candidate for protein vaccine. However, genetically engineered subunit vaccines often exhibit low immunogenicity, making it necessary to use adjuvants to enhance their effectiveness.

CpG ODNs are oligodeoxynucleotides that have been modified by non-methylation based on cytosine-phosphate-guanine (CpG) dinucleotide sequences. CpG 1018 was approved by the United States Food and Drug Administration as the world’s first adjuvant for hepatitis B vaccines. CpG ODNs hold significant potential as immunotherapeutic agents in the treatment of infectious diseases, allergies, and cancers in humans (23–25). Based on structural differences and immune-inducing properties, CpG ODN can be categorized into four types: A, B, C, and P (26–28). B-type CpG ODN induces differentiation in plasmacytoid dendritic cells (pDCs) and produces tumor necrosis factor α (TNF-α), acting as a strong activator of B cells, which, in turn, secrete a large number of immunoglobulins, interleukin (IL)-6, IL-10, and IL-12. However, its ability to activate NK cells is not as effective as that of A-type (29). C-type CpG ODN activates both pDCs and B cells. Double-palindromic P-type CpG ODN demonstrates a robust capacity to induce type I interferons and stimulates cytokine production when administered *in vivo* (28). B-type CpG ODN, recognized as an effective Th1-type adjuvant, is commonly utilized in vaccine research (30).

MF59, an oil-in-water adjuvant, is extensively used in human influenza vaccines due to its safety and effectiveness. Vaccines with MF59 adjuvant tend to primarily induce Th2 immune responses. However, studies have found that adding TLR9 agonists or TLR4 agonists to MF59 adjuvant vaccines can induce stronger Th1 cell immune responses, characterized by increased IgG2a titers, interferon-gamma (IFN-γ) secretion, and antibody titer (31–33). In this study, we screened CpG ODNs *in vitro* for their stimulatory activity in pigs. The selected CpG ODN was then combined with MF59 as a compound adjuvant to examine the immunoenhancing effects on spike subunit vaccines of PEDV through mouse and piglet vaccination trials.

## 2 Materials and methods

### 2.1 Cells and viruses

Vero cells were cultured in a humidified air containing 5% CO_2_ incubator at 37°C using Dulbecco’s Modified Eagle Medium (DMEM, Cytiva, USA) supplemented with 10% fetal bovine serum (FBS; Gibco, USA) as well as 100 U/mL penicillin and 10 μg/mL streptomycin for growth support. Sf9 and Hi5 cells were cultured in a constant temperature shaker incubator using IB905 serum-free medium (Yishengke, China). The PEDV strain was preserved by Yangling Carey Biotechnology Co., Ltd.

### 2.2 CpG ODNs

CpG1, CpG2, CpG3, CpG4, and CpG5 are B-type CpG ODNs with different sequences. These CpG ODNs are designed based on BW006, which has previously been shown to have stimulatory activity (34). For *in vitro* screening of CpG ODNs, BW006 and a meaningless sequence of GC were used as positive and negative controls, respectively. All CpG ODNs used in this study were provided by JSIAMA (Nanjing, China; **Table 1**) and were diluted in sterile endotoxin-free water.

**Table 1:** Synthesis sequence of CpG ODNs.

Name	Sequence
CpG1	5’-T-C-G-A-C-G-T-T-C-G-T-C-G-T-T-C-G-T-T-G-T-T-C-3’
CpG2	5’-T-T-G-A-C-G-T-T-C-G-T-C-G-T-T-C-G-T-C-G-T-T-C-3’
CpG3	5’-T-C-G-A-C-G-T-T-C-G-T-C-G-T-T-T-G-T-C-G-T-T-C-3’
CpG4	5’-T-C-G-A-C-G-T-T-C-G-T-T-G-T-T-C-G-T-C-G-T-T-C-3’
CpG5	5’- T-C-G-T-C-G-T-T-G-T-C-G-T-T-T-T-G-T-C-G-T-T-C-3’
GC	5’-T-G-G-C-C-A-A-G-C-T-T-G-G-G-C-C-C-C-T-T-G-C-A-A-G-G-G-C-C-3’
BW006	5’-T-C-G-A-C-G-T-T-C-G-T-C-G-T-T-C-G-T-C-G-T-T-C-3’

### 2.3 Lymphocyte proliferation assay

PBMCs from unvaccinated piglets were prepared by density gradient centrifugation. The isolated PBMCs were then suspended in Roswell Park Memorial Institute (RPMI) 1640 medium supplemented with 10% FBS. The cell density was adjusted to 4×10^6^ cells/mL and 50 μL of the suspension was seeded in each well of a 96-well plate. Subsequently, 50 μL of different concentrations of CpG, with GC as a negative control and BW006 as a positive control, were added to the wells. Simultaneously, an unstimulated control was established. The plates were incubated in a 5% CO_2_ incubator at 37°C for 72 hours. Afterward, 10 μL Cell Counting Kit 8 (CCK-8; DOJINDO, Japan) was added and incubated for another 4 hours. The absorbance at OD450 nm was measured using a microplate reader. Lymphocyte proliferation results are expressed as the stimulation index (SI), which is defined as the mean of the experimental data divided by the mean of the unstimulated control.

For the antigen-specific cell proliferation assay, mouse spleen lymphocytes were isolated from day 21 of inoculation and stimulated with 5 ug/mL PEDV S protein, with other steps as above.

### 2.4 Enzyme-linked immunospot assay

The ELISpot Flex: Porcine IFN-γ (ALP; Mabtech, USA) was used for conducting ELISPOT assays. Briefly, a Millipore 96-well polyvinylidene difluoride (PVDF) ELISPOT plate was treated with 35% ethanol (20 μL) for 1 minute. Afterward, the plate was washed five times with sterile water, and each well was incubated with 10 μg/mL of pIFN-γ-1 overnight at 4–8°C. Following another series of washes with phosphate-buffered saline (PBS; five times), the plate was exposed to RPMI 1640 (200 μL), which was then incubated for 30 minutes at room temperature. Subsequently, the plate was emptied, and CpG and controls (50 μL each) were added, followed by the addition of PBMCs (50 μL) at a cell density of 4×10^6^ cells/mL. The plate was then cultured in a 37°C and 5% CO_2_ incubator for 24 hours. After another series of washes with PBS (five times), the plate was incubated with 100 μL detection antibody of P2C11-biotin (0.5 μg/mL, Mabtech, USA) for 2 hours at room temperature. The plate was again washed with PBS (five times), and streptavidin-ALP (100 μL) was added, followed by incubation for 1 hour at room temperature. Finally, the plate was washed with PBS (five times), and BCIP/NBT-plus solution (100 μL) was added to develop the spots until they were distinctly visible (5–30 minutes).

### 2.5 Design, expression, purification of PEDV spike antigens

The spike gene of PEDV strain CT (GenBank Accession Number: MK539948.2) was utilized as the basis for designing the spike structure. The codons were specifically optimized for expression in insect cells. To modify the PEDV spike protein, the S1076P and L1077P mutations were introduced, resulting in the 2P mutation. In addition, a total of 64 residues were deleted from the C-terminal region, and the T4 fibritin trimerization element was fused to the C-terminal. Furthermore, a His8 tag with TEV protease was genetically attached to the N-terminal of the S protein. All vectors were expressed using the baculovirus insect cell expression system. Briefly, the density of sf9 cells was fine-tuned to 1.0 × 10^6^ cells/mL and then seeded onto 6-well cell culture plates (2 mL per well). The cells were incubated at 27°C for 30 minutes to permit adherence. Then, 100 µL of IB905 serum-free medium (Yishengke, China), 3 µg of the recombinant vector, and 3 µL of linearized bacmid were carefully combined with 5 µL of transfection reagent (Sunma, China). The mixture was left at room temperature for 20 minutes before being introduced into the 6-well cell culture plate containing sf9 cells and cultured at 27°C for 5 days to harvest the baculovirus virus. The baculovirus virus supernatant was inoculated with 1 multiplicity of infection (MOI) into 2.0 × 10^6^ cells/mL Hi5 cells to express the protein. The express supernatant was collected and the protein was purified by filtration through a 0.45-μm filter membrane and by using a Ni–NTA column (Qianchun, China), eluting with the buffer (50 mM Tris, 200 mM NaCl, 500 mM imidazole, pH 7.4). The purified protein was stored aseptically after dialysis in the preservation solution (50 mM Tris, 200 mM NaCl, pH 7.4).

The appropriate volume of 5× loading buffer was added to the purified protein, followed by mixing. The mixture was heated at 100°C for 10 minutes. Afterwards, the sample underwent sodium dodecyl sulfate–polyacrylamide gel electrophoresis (SDS-PAGE) at 140 V on a 10% polyacrylamide gel for 60 minutes. After the separation, one of the gel was stained with Coomassie Brilliant Blue (Beyotime, China) for 10 minutes and then destained with water before documentation was conducted. The other gel was transferred onto a PVDF membrane (Thermo, USA) at 100 V for 90 minutes. The PVDF membrane was blocked with 5% skim milk powder at 37°C for 2 hours, and then incubated with a 1,000-fold dilution of PEDV monoclonal antibody (QIANXUN, China) at 37°C for 60 minutes. The PVDF membrane was washed five times with PBS containing Tween 20 (PBST). Subsequently, the PVDF membrane was incubated at 37°C for 40 minutes with a 5,000-fold dilution of horseradish peroxidase (HRP)-conjugated goat anti-mouse IgG (H + L) (Proteintech, China). Following this, the PVDF membrane was again washed five times with PBST. For the detection of proteins, an ECL chromogenic solution (Dining, China) was used in the gel imaging system.

### 2.6 Animal experimental design

All animal experiments followed Northwest A&F University guidelines and were approved by the Institutional Animal Care and Use Committee (IACUC). To ensure animal welfare, the experiment conscientiously complied with the 3R principle. To initially investigate the immune effect of selected CpG5 with good stimulatory activity on S subunit vaccines of PEDV, we conducted a mouse vaccination study. Female BALB/c mice aged 6–8 weeks were obtained from Xi’an Yifengda Biotechnology Co., Ltd. The mice were divided into different groups: CpG5 and MF59 compound adjuvant group, MF59 adjuvant group, and control group with S protein alone and PBS (**Table 2**). Each group consisted of 6 mice. The adjuvant group vaccines were prepared by gently mixing PEDV S protein and adjuvant at a 1:1 volume ratio. For the different doses of CpG5 compound adjuvant groups, MF59 adjuvant was first formulated with different concentrations of CpG5 to create the compound adjuvants, which were then formulated with S protein to prepare the vaccines. The mice were immunized with subcutaneous injections of 200 µL on two separate days: days 0 and 14. Serum samples were collected from the mice on three different days: days 14, 21, and 42.

**Table 2:** Mouse immunity information.

Group	Antigen dose	Adjuvant 1	Adjuvant 2
PBS	none	none	none
S	20 μg	none	none
S/MF59	20 μg	MF59	none
S/MF59 + 5 μg CpG5	20 μg	MF59	5 μg CpG5
S/MF59 + 10 μg CpG5	20 μg	MF59	10 μg CpG5
S/MF59 + 20 μg CpG5	20 μg	MF59	20 μg CpG5
S/MF59 + 50 μg CpG5	20 μg	MF59	50 μg CpG5

For piglet vaccination, 30-day-old piglets were sourced from Tongchuan Lianyi Ecological Agriculture Co., Ltd with negative PEDV antigens and antibodies. Each group consisted of 4 piglets. The vaccines group included S/CpG5, S/MF59, and S/MF59 + CpG5. The negative control group was given PBS. The vaccines were formulated with 100 μg of PEDV S protein and adjuvant at a volume ratio of 1:1. The dosage of CpG5 is 100 ug per piglet. The piglets in the study received intramuscular injections of 2 mL for immunization on two separate days: day 0 and 14. Serum samples were collected from the piglets on three different days: days 14, 21, and 42.

### 2.7 Enzyme-linked immunosorbent assay

ELISA was performed to detect anti-S IgG and IgG isotypes antibody in mouse or pig serum samples. The S protein was diluted with 0.1 mol/L carbonate/bicarbonate buffer (pH 9.6) to a concentration of 1 μg/μL and coated on 96-well plates at 100 μL per well. Afterward, the plates were incubated at 4°C overnight before being washed four times with PBST. Subsequently, the coated plates were blocked with 5% skimmed milk powder at 37°C for 2 hours, washed four times with PBST, and gently patted dry. The serum samples were serially twofold diluted with 1% bovine serum albumin solution and 100 μL per well was added to the coated plates, followed by incubation at 37°C for 1 hour and then washed four times with PBST before being carefully patted dry. The next step involved the addition of 100 μL HRP-conjugated goat anti-mouse IgG/IgG1/IgG2a or goat anti-swine IgG in a 5000-fold dilution (Proteintech, China) followed by incubation at 37°C for 40 minutes, washed again four times with PBST, and carefully patted dry. Finally, 100 μL 3,3′,5,5′-Tetramethylbenzidine chromogenic solution (TMB, Beyotime, China) was introduced and incubated at 37°C for 10 minutes in the dark, after which the 50 μL stop solution of 2M H_2_SO_4_ was added, and absorbance values were measured at OD450 nm.

Splenic lymphocytes were isolated and resuspended in RPMI 1640 supplemented with 10% FBS (Gibco, USA). The cell density was adjusted to 4 × 10^6^ cells/mL and seeded into 96-well plates at a volume of 100 μL per well. PEDV S protein was added at a final concentration of 5 μg/mL, incubated in a 37°C, 5% CO_2_ incubator for 24 hours and then the supernatant was collected for detection. The concentrations of mouse IFN-γ, IL-2, IL-4, and IL-10 (mlBio, China) were detected by ELISA according to the kit manufacturer’s instructions.

### 2.8 Neutralization assay

A neutralization test was used to detect serum neutralizing antibody titer. mouse and piglet serum samples were inactivated in a water bath at 56°C for 30 minutes. Briefly, 2 × 10^4^ Vero cells were seeded into a 96-well cell culture plate and cultured in a 37°C, 5% CO_2_ incubator for 24 hours in advance. The inactivated serum was serially diluted twofold and mixed with 200 TCID_50_/100 μL of PEDV at a 1:1 volume ratio and incubated at 37°C for 1 hour. The mixture was added to a 96-well cell culture plate that had been washed twice with PBS and then cultured for 2 hours. The medium was then discarded, 100 μL of fresh DMEM containing 8 μg/mL trypsin was added and continue cultured for another 3–5 days. Cytopathic effects were recorded every day, and neutralizing antibody titers were finally calculated using the Reed-Muench method.

### 2.9 Negative-stain electron microscopy

The purified protein was diluted to a concentration of 0.2 mg/mL and applied onto carbon-coated copper grids. The grids were then incubated at room temperature for 5 minutes. Subsequently, the grids were immersed in PBS, and any excess liquid was removed using filter paper. After staining with phosphotungstic acid for 2 minutes, any remaining liquid was removed using filter paper again. Finally, the examination of the samples was conducted through the utilization of an HT7800 transmission electron microscope (Hitachi, Japan) at 80 kV.

### 2.10 Flow cytometry analysis

PBMCs were collected from piglets in each group at 21 days post-immunization. CD3-FITC mAb, CD4-PerCP-Cy5.5 mAb, and CD8α-PE mAb antibodies (BD, USA) were utilized for staining of PBMCs at appropriate concentrations. Flow cytometry analyses were performed using FACSAria™ III flow cytometer (BD, USA), and data were analyzed with the software of FlowJo version 10.6.2 (BD, USA).

### 2.11 Statistical analysis

Data were presented as mean ± standard error of the mean. One-Way ANOVA and Tukey multiple comparison test were used for significance analysis. The statistical software of GraphPad Prism version 9.0.0 was utilized for all data analysis. Statistical significance was defined as *p <0.05, **p <0.01, ***p <0.001, ****p <0.0001, while “ns” indicates non-significance. The asterisk above the horizontal line indicates a comparison between the two groups.

## 3 Result

### 3.1 Porcine PBMC proliferation studies

**Figure 1:**
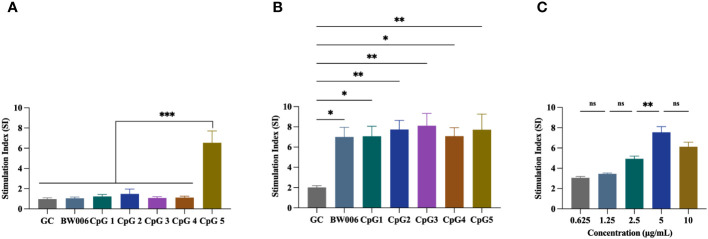
Stimulation of porcine peripheral blood mononuclear cells (PBMCs) proliferation by CpG oligodeoxynucleotides (ODNs). **(A)** PBMCs proliferation was stimulated with 5 μg/mL CpG ODNs. **(B)** PBMCs proliferation was stimulated with 10 μg/mL CpG ODNs. **(C)** Dose-dependent study of CpG5. The data was expressed as the means ± SEM (n = 5; ns, not significant, *P < 0.05, **P < 0.01, ***P < 0.001).

The proliferation activity of PBMCs from unvaccinated piglets was assessed using a CCK-8 assay. Five B-type CpG ODNs (CpG1, CpG2, CpG3, CpG4, and CpG5) as well as negative control (GC) and positive control (BW006) were tested at two concentrations. At a concentration of 5 μg/mL, CpG1, CpG2, CpG3, and CpG4 exhibited no proliferation activity of PBMCs (stimulation index [SI] < 2), while CpG5 significantly increased PBMCs proliferation (SI > 6; P < 0.001; **Figure 1A**). At a concentration of 10 μg/mL, all CpG ODNs demonstrated a significant ability to stimulate PBMCs proliferation (SI > 6) compared to the GC control group (**Figure 1B**). No significant difference was observed in stimulatory activity among the experimental groups at high concentration (**Figure 1B**). A dose-dependent study was also conducted for CpG5, revealing that stimulatory activity increased with escalating doses within the range of 0.625–5 mg/mL, but no longer increased above 5 mg/mL (**Figure 1C**).

### 3.2 Evaluation of IFN-γ secretion by ELISPOT

**Figure 2:**
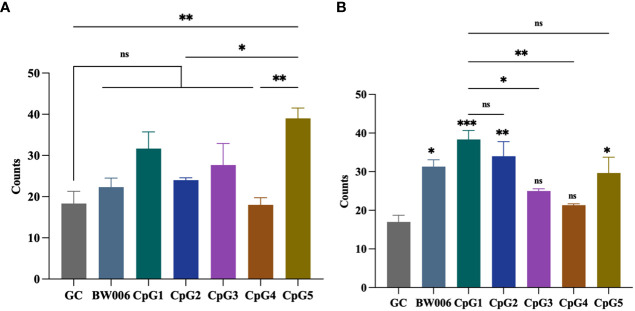
CpG oligodeoxynucleotides (ODNs) stimulate the secretion of interferon-gamma (IFN-γ) from porcine peripheral blood mononuclear cells (PBMCs). **(A)** Stimulation with 5 μg/mL of CpG ODNs. **(B)** Stimulation with 10 μg/mL of CpG ODNs. The data was expressed as the means ± SEM (n = 5; ns, not significant, *P < 0.05, **P < 0.01, ***P < 0.001). The symbols above the bar chart represent statistical significance compared to the GC group.

The ELISPOT assay was performed to determine the number of spots stimulated to secrete IFN-γ from PBMCs of unvaccinated piglets. Five CpG ODNs, along with negative control (GC) and positive control (BW006), were tested at concentrations of 5 μg/mL and 10 μg/mL. When CpG ODNs were administered at a concentration of 5 μg/mL, only CpG5 showed the activity of inducing IFN-γ secretion compared to the GC group (**Figure 2A**). When the concentration of CpG ODNs increased to 10 μg/mL, CpG3 and CpG4 still did not induce IFN-γ secretion, while the other groups significantly induced IFN-γ secretion compared to the GC group (**Figure 2B**). The activity of CpG1 in inducing IFN-γ secretion was significantly higher than that of CpG3 and CpG4, but there was no significant difference compared to CpG5 (**Figure 2B**).

### 3.3 PEDV antigen purification and characterization

**Figure 3:**
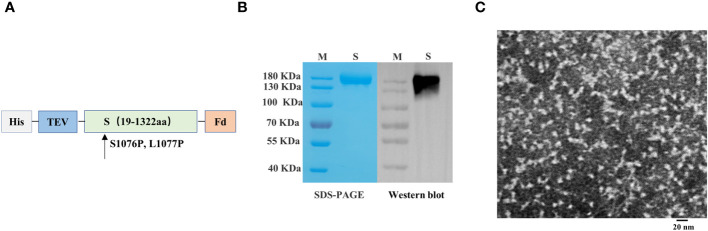
Preparation and characterization of porcine epidemic diarrhea virus (PEDV) antigens. **(A)** Schematics illustrating the structure of PEDV spike protein. **(B)** Analysis of PEDV spike protein by sodium dodecyl sulfate–polyacrylamide gel electrophoresis and western blotting. **(C)** Transmission electron microscope image of PEDV spike protein.

We engineered a PEDV spike trimer by incorporating the Fd trimer domain, removing 64 residues at the C terminus, and introducing the 2P mutation (S1076P/L1077P; **Figure 3A**). The recombinant S protein of PEDV was produced using the baculovirus insect expression system. The His-affinity purified protein attained satisfactory purity and demonstrated reactivity with the PEDV monoclonal antibody in western blotting analysis (**Figure 3B**). Transmission electron microscopy showed that the spike protein of PEDV exhibited a unique trimer structure (**Figure 3C**).

### 3.4 Humoral immune response in mice

**Figure 4:**
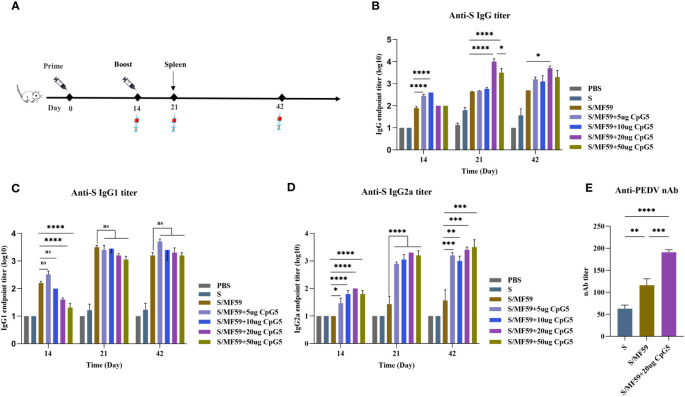
Humoral immune response of porcine epidemic diarrhea virus (PEDV) subunit vaccines in BALB/c mice. **(A)** Schematic of the immunization procedure for BALB/c mice. **(B)** The endpoint titer of PEDV IgG was detected at each time point in BALB/c immunized mice. **(C)** The endpoint titer of PEDV IgG1 was detected at each time point in BALB/c immunized mice. **(D)** The endpoint titer of PEDV IgG2a was detected at each time point in BALB/c immunized mice. **(E)** Measurement of PEDV neutralizing antibody titer in immunized BALB/c mice. The data was expressed as the means ± SEM (n = 6; ns, not significant, *P < 0.05, **P < 0.01, ***P < 0.001, ****P < 0.0001).

We screened CpG5 with good stimulatory effects *in vitro* and combined it with MF59 as a compound adjuvant. To assess the humoral immunoenhancing effects of the CpG5 compound adjuvant on PEDV subunit vaccines, we prepared MF59, and different doses of CpG5 (5 μg, 10 μg, 20 μg, and 50 μg) compound adjuvant. The negative control group received PBS and S protein alone as an unadjuvanted control. Subcutaneous injections were administered to BALB/c mice according to the immunization schedule (**Figure 4A**). The results indicated that the IgG antibody titer in the low-dose CpG5 (5 μg, 10 μg) compound adjuvant groups was significantly higher than that in the MF59 adjuvant group at 14 days post-immunization, but there was no significant difference at 21 days (**Figure 4A**). Conversely, IgG antibody titer of the high-dose CpG5 (20 μg, 50 μg) compound adjuvant groups was significantly augmented compared to the MF59 adjuvant group at 21 days (**Figure 4A**). Moreover, at day 42, the IgG antibody titer of the 20 μg CpG5 compound adjuvant group still remained significantly higher than the MF59 adjuvant group and remained at a consistently high level (**Figure 4A**).

For a more comprehensive understanding of the immune responses during immunization, an in-depth analysis of the induced antibody IgG subtypes was conducted. The findings revealed that the IgG1 antibody titer induced by MF59 adjuvant and low-dose CpG5 (5 μg, 10 μg) compound adjuvant groups exceeded those of high-dose CpG5 (20 μg, 50 μg) compound adjuvant groups at 14 days post-immunization, but there was no significant difference among the adjuvant groups after the second dose (**Figure 4C**). On the other hand, significantly greater level of IgG2a antibody was observed within the CpG5 compound adjuvant groups at all time points (**Figure 4D**). Interestingly, we found that the low-dose CpG5 (5 μg, 10 μg) compound adjuvant groups were more dominant in inducing IgG in the early stages, possibly due to the fact that the low-dose CpG5 and MF59 adjuvant synergistically biased towards the Th2 immune response, while when CpG5 accumulated to a certain dose, mainly resulted in a Th1 immune response characterized by elevated IgG2a antibody titer (**Figures 4B–D**).

In addition to the magnitude of antibody production, the neutralizing capability of antibody production stands as another pivotal factor influencing the quality of immunity. To further evaluate the immunoenhancing effects of the CpG5 compound adjuvant on PEDV subunit vaccines, neutralizing antibody titer was assessed. The neutralizing antibody titer of the S protein, the MF59 adjuvant, and the 20 ug CpG5 compound adjuvant groups was primarily tested at 21 days post-immunization. The outcomes demonstrated that the adjuvant groups had a significantly higher neutralizing antibody titer compared to the non-adjuvanted S group (**Figure 4E**). Moreover, concurrent with significant enhancement of neutralizing antibody titer in 20 ug CpG5 compound adjuvant group compared to MF59 adjuvant group (**Figure 4E**). In summary, the combination of MF59 adjuvant with CpG5 substantially enhanced the humoral immune response to PEDV subunit vaccines in mice.

### 3.5 T cell immune response in mice

**Figure 5:**
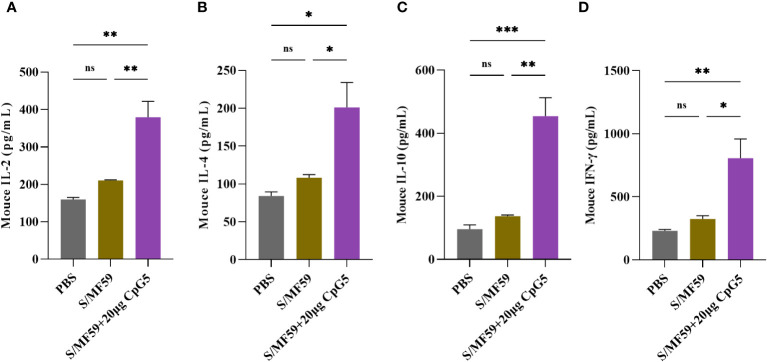
Evaluation of the cellular immune response of a porcine epidemic diarrhea virus (PEDV) subunit vaccine in mice. **(A)** Concentration of interleukin (IL)-2 was determined using enzyme-linked immunosorbent assay (ELISA). **(B)** Concentration of IL-4 was determined using ELISA. **(C)** Concentration of IL-10 was determined using ELISA. **(D)** Concentration of interferon-gamma (IFN-γ) was determined using ELISA. Splenic lymphocytes from immunized mice were isolated at day 21 post-immunization. The data was expressed as the means ± SEM (n = 3; ns, not significant, *P < 0.05, **P < 0.01, ***P < 0.001).

To investigate the effects of CpG5 compound adjuvant on the cellular immune response triggered by PEDV subunit vaccines, spleen lymphocytes from mice were isolated at the 21 days post-immunization. 5 μg/mL S protein was used to ex-vivo stimulation of spleen lymphocytes from three groups, PBS, MF59 and CpG5 compound adjuvant to study cytokine secretion. Our findings demonstrated that the CpG5 compound adjuvant group significantly increased the secretion of IL-2 (p < 0.01; **Figure 5A**), IL-4 (p < 0.05; **Figure 5B**), IL-10 (p < 0.01; **Figure 5C**), and IFN-γ (p < 0.05; **Figure 5D**) cytokines compared to the MF59 group alone. The IFN-γ/IL-4 ratio, commonly used to assess Th1 or Th2 immune responses, was approximately 4 in CpG5 compound adjuvant group, indicating a robust Th1 immune response induced by the combined CpG5.

### 3.6 Antigen-specific spleen lymphocytes proliferation is encouraged by CpG

**Figure 6:**
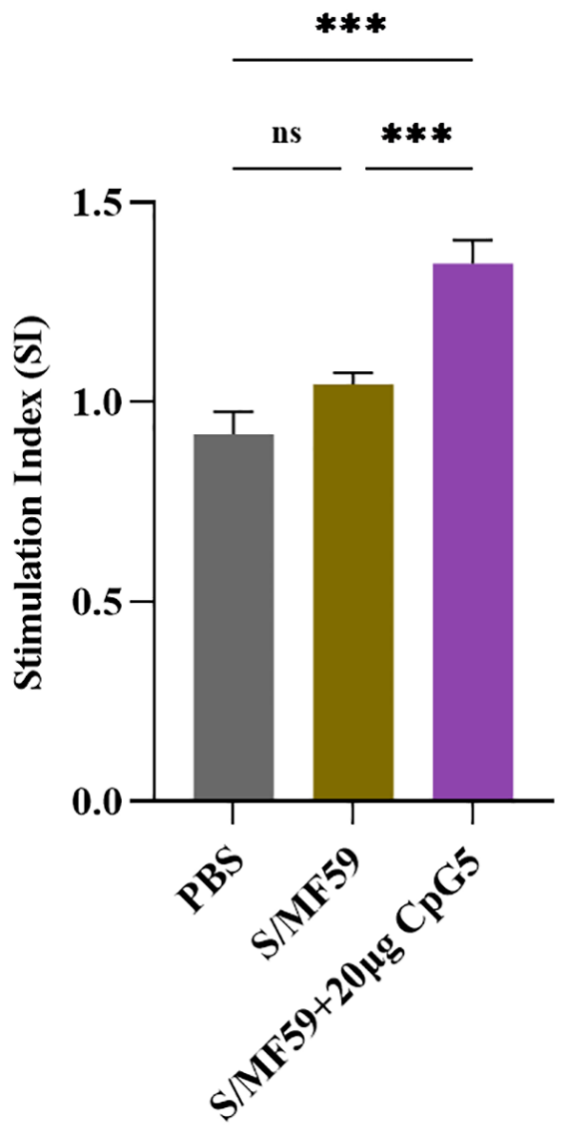
Antigen-specific lymphocyte proliferation. Lymphocyte proliferation was stimulated by spike protein of porcine epidemic diarrhea virus. Splenic lymphocyte from immunized mice were isolated at day 21 post-immunization. The data was expressed as the means ± SEM (n = 3; ns, not significant, ***P < 0.001).

To further explore antigen-specific splenic lymphocyte proliferation, mouse splenic lymphocytes were isolated at the 21 days post-immunization. These lymphocytes were then stimulated with the S protein of PEDV at a final concentration of 5 μg/mL and detected using the CCK-8 assay. The results revealed that the CpG5 compound adjuvant group displayed significantly heightened lymphocyte proliferation compared to MF59 adjuvant and the PBS control group (**Figure 6**).

### 3.7 Humoral immune response in piglets

**Figure 7:**
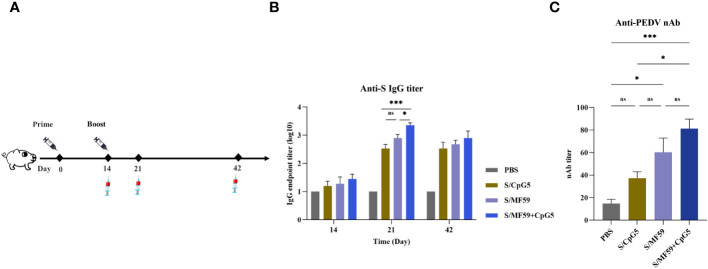
Humoral immune response of porcine epidemic diarrhea virus (PEDV) subunit vaccine in piglets. **(A)** Schematic of the piglet immunization procedure. **(B)** The endpoint titer of PEDV IgG was detected at each time point in immunized piglets. **(C)** Detection of PEDV-neutralizing antibody titers in immunized piglets. The data was expressed as the means ± SEM (n = 4; ns, not significant, *P < 0.05, ***P < 0.001).

To further evaluate the impact of the CpG5 compound adjuvant on PEDV subunit vaccines, we conducted a study using a piglet vaccine model. A series of immunological procedures were administered via intramuscular injection to 30-day-old piglets (**Figure 7A**). The negative control group received PBS. The results revealed that the titer of IgG antibody in CpG5 compound adjuvant group was significantly higher than compared to the CpG5 and MF59 adjuvant groups alone at 21 days post-immunization (**Figure 7B**). There was no significant difference between the CpG5 and MF59 adjuvant group (**Figure 7B**). Further neutralization assay demonstrated that the neutralizing antibody titer in the CpG5 compound adjuvant group was significantly enhanced compared to the PBS control group at 21 days post-immunization (**Figure 7C**). In summary, the co-administration of MF59 and CpG5 significantly augmented the humoral immune response elicited by the PEDV subunit vaccine.

### 3.8 CpG significantly increases the proportion of CD8+ T cells

**Figure 8:**
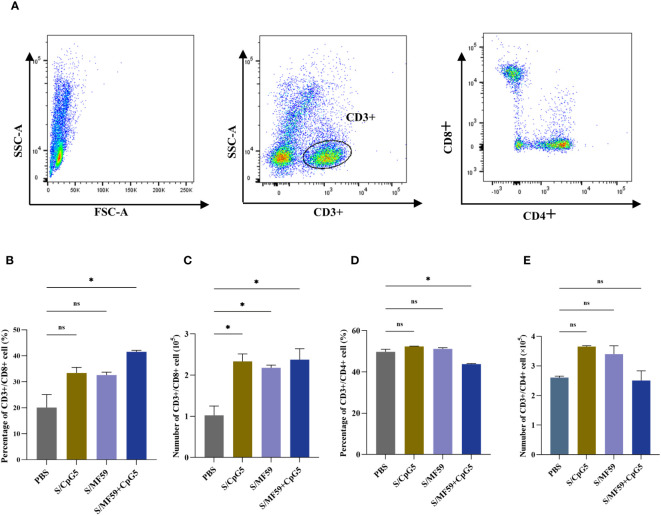
T cell responses in immunized piglets. **(A)** Representative gating strategy for evaluating T cells. **(B)** Proportion of CD3+/CD8+ T cells in PBMCs of different groups. **(C)** Number of CD3+/CD8+ T cells in PBMCs of different groups. **(D)** Proportion of CD3+/CD4+ T cells in PBMCs of different groups. **(E)** Number of CD3+/CD4+ T cells in PBMCs of different groups. The data was expressed as the means ± SEM (n = 4; ns, not significant, *P < 0.05).

To investigate the immunoenhancing effects of the compound adjuvant on the cellular immune response to the PEDV subunit vaccine in piglets, PBMCs were isolated and analyzed using flow cytometry at 21 days post-immunization. Representative gating strategies were used for flow analysis of T lymphocytes (**Figure 8A**). The results demonstrated a significant enhancement in the proportion and absolute number of CD8+ T cells in the MF59+CpG5 adjuvant group compared to the PBS control group (**Figures 8B, C**). Similarly, the absolute number of CD8+ T cells in the CpG5 and MF59 adjuvant groups alone showed a increase compared to the PBS control group, although there was no significant difference in proportion (**Figures 8B, C**). Furthermore, the percentage of CD4+ T cells in the MF59+CpG5 adjuvant group was lower than that in the PBS control group, while there was no significant difference in the absolute number of CD4+ T cells among other groups (**Figures 8D, E**). These findings suggest that the MF59+CpG adjuvant group stimulates a cellular immune response primarily mediated by CD3+/CD8+ T cells in piglets.

## 4 Discussion

B-type CpG ODNs encode multiple CpG motifs on a phosphodiester backbone with a linear structure. The basic sequence structure consists of purine-pyrimidine-C-G-pyrimidine-pyrimidine. Previous research identified 5′-GTCGTT-3′ as a human structural motif (35), while 5′-GACGTT-3′ is considered a mouse structural motif (36). Studies have documented that pigs respond more strongly to the B-type CpG motif 5′-ATCGAT-3′ (37). B-type CpG has been shown to enhance antigen-induced humoral and cell-mediated immune responses (38, 39). In this study, we evaluated five pig-specific B-type CpG ODNs *in vitro*. The results showed that CpG5 significantly stimulated PBMCs proliferation and IFN-γ secretion at a concentration of 5 μg/mL, however, CpG3 and CpG4 did not stimulate IFN-γ secretion at either low or high concentrations. Although CpG1 can significantly stimulate PBMCs proliferation and IFN-γ secretion at high concentrations, it is still not significantly different from CpG5. Additionally, the stimulatory activity of CpG was found to be dose-dependent.

CpG can enhance immune responses to antigens, amplify the immune effects of various vaccine types, induce a Th1 immune response, and is regarded as an effective and safe vaccine adjuvant (30, 40). CpG has been extensively researched and employed in the treatment of human tumors and as a vaccine adjuvant (41). In recent years, CpG has garnered significant attention in animal vaccine research, including vaccines for swine (42–44), cattle (45–47), and chicken (48–50), among others. In this study, the use of CpG5 and MF59 compound adjuvant markedly augmented both humoral and cellular immune responses to the PEDV subunit vaccine. Moreover, it elicited a higher rate of antigen-specific lymphocyte proliferation in a mouse vaccination model, underscoring the distinct advantages of CpG5. CpG is recognized to provoke robust cytotoxic T lymphocyte activity across various hosts (51–53). Similarly, CpG5 effectively initiated a more potent cytotoxic function within the piglet immune system and led to a greater proportion of CD8+ T lymphocytes in piglet vaccination models. Furthermore, our findings indicate that CpG5, when combined, significantly enhanced and expedited the humoral immune response in piglets to the PEDV subunit vaccine.

The MF59 and CpG compound adjuvant had demonstrated their ability to amplify T cell responses and bolster viral control in subunit vaccines targeting the bovine viral diarrhea virus (45). In addition to inhibiting tumor growth, the MF59 and CpG compound adjuvant also triggered a Th1 immune response through the mucin 1 (MUC1)-maltose-binding protein (MBP) (MM) vaccine, leading to increased survival time in both preventive and therapeutic mice (54). This study demonstrated that the combination of MF59 and CpG5 substantially augmented both humoral and cellular immune reactions to the PEDV vaccine in mouse and piglet vaccination models. This observation implies that this compound adjuvant possesses considerable potential as a vaccine adjuvant. Past research has also indicated that combining CpG with ISA 206 adjuvant can boost the immune response to the recombinant foot-and-mouth disease vaccine (47). Additionally, a novel PELC and CpG compound adjuvant has been proven to trigger elevated neutralizing antibody levels in the serum and enhance the immune response against H7N9 avian influenza in mice (55). Hence, employing CpG as a combination adjuvant can induce an earlier, more potent, and enduring immune response, marking its potential for diverse applications.

## Data availability statement

The raw data supporting the conclusions of this article will be made available by the authors, without undue reservation.

## Ethics statement

The animal study was approved by Northwest A&F University Institutional Animal Care and Use Committee (IACUC). The study was conducted in accordance with the local legislation and institutional requirements.

## Author contributions

YW: Writing – review & editing, Methodology, Project administration, Writing – original draft, Formal analysis. SL: Writing – review & editing, Writing – original draft, Data curation, Investigation, Visualization. BL: Validation, Writing – review & editing, Investigation. XS: Methodology, Writing – review & editing, Visualization. QP: Writing – review & editing, Data curation. YXZ: Writing – review & editing, Validation. JL: Writing – review & editing, Formal analysis. YQZ: Writing – review & editing, Data curation. JW: Conceptualization, Writing – review & editing, Supervision. LL: Conceptualization, Resources, Writing – review & editing. ED: Conceptualization, Funding acquisition, Resources, Writing – review & editing.

## References

[1] Wood EN. An apparently new syndrome of porcine epidemic diarrhoea. Veterinary Rec (1977) 100(12):243–4. doi: 10.1136/vr.100.12.243 888300

[2] Pensaert MB de Bouck P. A new coronavirus-like particle associated with diarrhea in swine. Arch Virol (1978) 58(3):243–7. doi: 10.1007/BF01317606 PMC7086830 83132

[3] Sun RQ Cai RJ Chen YQ Liang PS Chen DK Song CX. Outbreak of porcine epidemic diarrhea in suckling piglets, China. Emerg Infect Dis (2012) 18(1):161–3. doi: 10.3201/eid1801.111259 PMC3381683 22261231

[4] Stevenson GW Hoang H Schwartz KJ Burrough ER Sun D Madson D. Emergence of Porcine epidemic diarrhea virus in the United States: clinical signs, lesions, and viral genomic sequences. J Vet Diagn Invest (2013) 25(5):649–54. doi: 10.1177/1040638713501675 23963154

[5] Vlasova AN Marthaler D Wang Q Culhane MR Rossow KD Rovira A. Distinct characteristics and complex evolution of PEDV strains, North America, May 2013-February 2014. Emerg Infect Dis (2014) 20(10):1620–8. doi: 10.3201/eid2010.140491 PMC4193278 25279722

[6] Masuda T Murakami S Takahashi O Miyazaki A Ohashi S Yamasato H. New porcine epidemic diarrhoea virus variant with a large deletion in the spike gene identified in domestic pigs. Arch Virol (2015) 160(10):2565–8. doi: 10.1007/s00705-015-2522-z PMC7087250 26162305

[7] Kim SH Lee JM Jung J Kim IJ Hyun BH Kim HI. Genetic characterization of porcine epidemic diarrhea virus in Korea from 1998 to 2013. Arch Virol (2015) 160(4):1055–64. doi: 10.1007/s00705-015-2353-y PMC7086719 25666198

[8] Diep NV Sueyoshi M Izzati U Fuke N Teh APP Lan NT. Appearance of US-like porcine epidemic diarrhoea virus (PEDV) strains before US outbreaks and genetic heterogeneity of PEDVs collected in Northern Vietnam during 2012-2015. Transbound Emerg Dis (2018) 65(1):e83–93. doi: 10.1111/tbed.12681 PMC7169849 28758349

[9] Gao Q Zheng Z Wang H Yi S Zhang G Gong L. The new porcine epidemic diarrhea virus outbreak may mean that existing commercial vaccines are not enough to fully protect against the epidemic strains. Front Vet Sci (2021) 8:697839. doi: 10.3389/fvets.2021.697839 34291104 PMC8287018

[10] Joyce MG Chen WH Sankhala RS Hajduczki A Thomas PV Choe M. SARS-CoV-2 ferritin nanoparticle vaccines elicit broad SARS coronavirus immunogenicity. Cell Rep (2021) 37(12):110143. doi: 10.1016/j.celrep.2021.110143 34919799 PMC8651551

[11] Brouwer PJM Brinkkemper M Maisonnasse P Dereuddre-Bosquet N Grobben M Claireaux M. Two-component spike nanoparticle vaccine protects macaques from SARS-CoV-2 infection. Cell (2021) 184(5):1188–200.e19. doi: 10.1016/j.cell.2021.01.035 33577765 PMC7834972

[12] Ma X Zou F Yu F Li R Yuan Y Zhang Y. Nanoparticle vaccines based on the receptor binding domain (RBD) and heptad repeat (HR) of SARS-coV-2 elicit robust protective immune responses. Immunity (2020) 53(6):1315–30.e9. doi: 10.1016/j.immuni.2020.11.015 33275896 PMC7687490

[13] Darricarrère N Pougatcheva S Duan X Rudicell RS Chou TH DiNapoli J. Development of a Pan-H1 influenza vaccine. J Virol (2018) 92(22):e01349-18. doi: 10.1128/JVI.01349-18 30185594 PMC6206494

[14] Kanekiyo M Wei CJ Yassine HM McTamney PM Boyington JC Whittle JR. Self-assembling influenza nanoparticle vaccines elicit broadly neutralizing H1N1 antibodies. Nature (2013) 499(7456):102–6. doi: 10.1038/nature12202 PMC8312026 23698367

[15] Marcandalli J Fiala B Ols S Perotti M de van der Schueren W Snijder J. Induction of potent neutralizing antibody responses by a designed protein nanoparticle vaccine for respiratory syncytial virus. Cell (2019) 176(6):1420–31.e17. doi: 10.1016/j.cell.2019.01.046 30849373 PMC6424820

[16] He L Chaudhary A Lin X Sou C Alkutkar T Kumar S. Single-component multilayered self-assembling nanoparticles presenting rationally designed glycoprotein trimers as Ebola virus vaccines. Nat Commun (2021) 12(1):2633. doi: 10.1038/s41467-021-22867-w 33976149 PMC8113551

[17] Bosch BJ van der Zee R de Haan CA Rottier PJ. The coronavirus spike protein is a class I virus fusion protein: structural and functional characterization of the fusion core complex. J Virol (2003) 77(16):8801–11. doi: 10.1128/JVI.77.16.8801-8811.2003 PMC167208 12885899

[18] Li F. Structure, function, and evolution of coronavirus spike proteins. Annu Rev Virol (2016) 3(1):237–61. doi: 10.1146/annurev-virology-110615-042301 PMC5457962 27578435

[19] Wrapp D McLellan JS. The 3.1-angstrom cryo-electron microscopy structure of the porcine epidemic diarrhea virus spike protein in the prefusion conformation. J Virol (2019) 93(23):e00923-19. doi: 10.1128/JVI.00923-19 31534041 PMC6854500

[20] Huang CY Draczkowski P Wang YS Chang CY Chien YC Cheng YH. *In situ* structure and dynamics of an alphacoronavirus spike protein by cryo-ET and cryo-EM. Nat Commun (2022) 13(1):4877. doi: 10.1038/s41467-022-32588-3 35986008 PMC9388967

[21] Kirchdoerfer RN Wang N Pallesen J Wrapp D Turner HL Cottrell CA. Stabilized coronavirus spikes are resistant to conformational changes induced by receptor recognition or proteolysis. Sci Rep (2018) 8(1):15701. doi: 10.1038/s41598-018-34171-7 30356097 PMC6200764

[22] Pallesen J Wang N Corbett KS Wrapp D Kirchdoerfer RN Turner HL. Immunogenicity and structures of a rationally designed prefusion MERS-CoV spike antigen. Proc Natl Acad Sci USA (2017) 114(35):E7348–e57. doi: 10.1073/pnas.1707304114 PMC5584442 28807998

[23] Søgaard OS Lohse N Harboe ZB Offersen R Bukh AR Davis HL. Improving the immunogenicity of pneumococcal conjugate vaccine in HIV-infected adults with a toll-like receptor 9 agonist adjuvant: a randomized, controlled trial. Clin Infect Dis (2010) 51(1):42–50. doi: 10.1086/653112 20504165

[24] Frank MJ Reagan PM Bartlett NL Gordon LI Friedberg JW Czerwinski DK. *In situ* vaccination with a TLR9 agonist and local low-dose radiation induces systemic responses in untreated indolent lymphoma. Cancer Discov (2018) 8(10):1258–69. doi: 10.1158/2159-8290.CD-18-0743 PMC6171524 30154192

[25] Hanagata N. CpG oligodeoxynucleotide nanomedicines for the prophylaxis or treatment of cancers, infectious diseases, and allergies. Int J Nanomedicine (2017) 12:515–31. doi: 10.2147/IJN.S114477 PMC5248940 28144136

[26] Verthelyi D Ishii KJ Gursel M Takeshita F Klinman DM. Human peripheral blood cells differentially recognize and respond to two distinct CPG motifs. J Immunol (Baltimore Md 1950). (2001) 166(4):2372–7. doi: 10.4049/jimmunol.166.4.2372 11160295

[27] Hartmann G Battiany J Poeck H Wagner M Kerkmann M Lubenow N. Rational design of new CpG oligonucleotides that combine B cell activation with high IFN-alpha induction in plasmacytoid dendritic cells. Eur J Immunol (2003) 33(6):1633–41. doi: 10.1002/eji.200323813 12778481

[28] Samulowitz U Weber M Weeratna R Uhlmann E Noll B Krieg AM. A novel class of immune-stimulatory CpG oligodeoxynucleotides unifies high potency in type I interferon induction with preferred structural properties. Oligonucleotides (2010) 20(2):93–101. doi: 10.1089/oli.2009.0210 20384481

[29] Vollmer J Weeratna R Payette P Jurk M Schetter C Laucht M. Characterization of three CpG oligodeoxynucleotide classes with distinct immunostimulatory activities. Eur J Immunol (2004) 34(1):251–62. doi: 10.1002/eji.200324032 14971051

[30] Bode C Zhao G Steinhagen F Kinjo T Klinman DM. CpG DNA as a vaccine adjuvant. Expert Rev Vaccines (2011) 10(4):499–511. doi: 10.1586/erv.10.174 21506647 PMC3108434

[31] Wack A Baudner BC Hilbert AK Manini I Nuti S Tavarini S. Combination adjuvants for the induction of potent, long-lasting antibody and T-cell responses to influenza vaccine in mice. Vaccine (2008) 26(4):552–61. doi: 10.1016/j.vaccine.2007.11.054 18162266

[32] Baudner BC Ronconi V Casini D Tortoli M Kazzaz J Singh M. MF59 emulsion is an effective delivery system for a synthetic TLR4 agonist (E6020). Pharm Res (2009) 26(6):1477–85. doi: 10.1007/s11095-009-9859-5 19255727

[33] Singh M Kazzaz J Ugozzoli M Baudner B Pizza M Giuliani M. MF59 oil-in-water emulsion in combination with a synthetic TLR4 agonist (E6020) is a potent adjuvant for a combination Meningococcus vaccine. Hum Vaccines Immunotherapeutics (2012) 8(4):486–90. doi: 10.4161/hv.19229 22832252

[34] Zhang X He P Hu Z Wang X Liang Z. Enhanced specific immune responses by CpG DNA in mice immunized with recombinant hepatitis B surface antigen and HB vaccine. Virol J (2011) 8(1):78. doi: 10.1186/1743-422X-8-7 21342531 PMC3050826

[35] Hartmann G Weeratna RD Ballas ZK Payette P Blackwell S Suparto I. Delineation of a CpG phosphorothioate oligodeoxynucleotide for activating primate immune responses in *vitro* and in vivo. J Immunol (Baltimore Md 1950) (2000) 164(3):1617–24. doi: 10.4049/jimmunol.164.3.1617 10640783

[36] Wang S Liu X Caulfield MJ. Adjuvant synergy in the response to hepatitis B vaccines. Vaccine (2003) 21(27-30):4297–306. doi: 10.1016/S0264-410X(03)00463-8 14505912

[37] Kamstrup S Verthelyi D Klinman DM. Response of porcine peripheral blood mononuclear cells to CpG-containing oligodeoxynucleotides. Vet Microbiol (2001) 78(4):353–62. doi: 10.1016/S0378-1135(00)00300-X 11182501

[38] Lipford GB Bauer M Blank C Reiter R Wagner H Heeg K. CpG-containing synthetic oligonucleotides promote B and cytotoxic T cell responses to protein antigen: a new class of vaccine adjuvants. Eur J Immunol (1997) 27(9):2340–4. doi: 10.1002/eji.1830270931 9341778

[39] Chiodetti AL Sánchez Vallecillo MF Dolina JS Crespo MI Marin C Schoenberger SP. Class-B cpG-ODN formulated with a nanostructure induces type I interferons-dependent and CD4(+) T cell-independent CD8(+) T-cell response against unconjugated protein antigen. Front Immunol (2018) 9:2319. doi: 10.3389/fimmu.2018.02319 30364187 PMC6192457

[40] Yang JX Tseng JC Yu GY Luo Y Huang CF Hong YR. Recent advances in the development of toll-like receptor agonist-based vaccine adjuvants for infectious diseases. Pharmaceutics (2022) 14(2):423. doi: 10.3390/pharmaceutics14020423 35214155 PMC8878135

[41] Shirota H Tross D Klinman DM. CpG oligonucleotides as cancer vaccine adjuvants. Vaccines (Basel) (2015) 3(2):390–407. doi: 10.3390/vaccines3020390 26343193 PMC4494345

[42] Zhang L Tian X Zhou F. Intranasal administration of CpG oligonucleotides induces mucosal and systemic Type 1 immune responses and adjuvant activity to porcine reproductive and respiratory syndrome killed virus vaccine in piglets in vivo. Int Immunopharmacol (2007) 7(13):1732–40. doi: 10.1016/j.intimp.2007.09.012 17996683

[43] Chen TH Chen CC Huang MH Huang CH Jan JT Wu SC. Use of PELC/CpG adjuvant for intranasal immunization with recombinant hemagglutinin to develop H7N9 mucosal vaccine. Vaccines (Basel) (2020) 8(2):240. doi: 10.3390/vaccines8020240 32455704 PMC7349964

[44] Dhakal S Ghimire S Renu S Ross KA Lakshmanappa YS Hogshead BT. Evaluation of CpG-ODN-adjuvanted polyanhydride-based intranasal influenza nanovaccine in pigs. Vet Microbiol (2019) 237:108401. doi: 10.1016/j.vetmic.2019.108401 31585639

[45] Wang S Yang G Nie J Yang R Du M Su J. Recombinant E(rns)-E2 protein vaccine formulated with MF59 and CPG-ODN promotes T cell immunity against bovine viral diarrhea virus infection. Vaccine (2020) 38(22):3881–91. doi: 10.1016/j.vaccine.2020.03.020 32280039

[46] Rankin R Pontarollo R Gomis S Karvonen B Willson P Loehr BI. CpG-containing oligodeoxynucleotides augment and switch the immune responses of cattle to bovine herpesvirus-1 glycoprotein D. Vaccine (2002) 20(23-24):3014–22. doi: 10.1016/S0264-410X(02)00216-5 12126915

[47] Ren J Yang L Xu H Zhang Y Wan M Liu G. CpG oligodeoxynucleotide and montanide ISA 206 adjuvant combination augments the immune responses of a recombinant FMDV vaccine in cattle. Vaccine (2011) 29(45):7960–5. doi: 10.1016/j.vaccine.2011.08.072 21872635

[48] Gunawardana T Ahmed KA Goonewardene K Popowich S Kurukulasuriya S Karunarathna R. Synthetic CpG-ODN rapidly enriches immune compartments in neonatal chicks to induce protective immunity against bacterial infections. Sci Rep (2019) 9(1):341. doi: 10.1038/s41598-018-36588-6 30674918 PMC6344490

[49] Fang L Zhen Y Su Q Zhu H Guo X Zhao P. Efficacy of CpG-ODN and Freund's immune adjuvants on antibody responses induced by chicken infectious anemia virus VP1, VP2, and VP3 subunit proteins. Poultry Sci (2019) 98(3):1121–6. doi: 10.3382/ps/pey475 30376069

[50] Lin SY Yao BY Hu CJ Chen HW. Induction of robust immune responses by cpG-ODN-loaded hollow polymeric nanoparticles for antiviral and vaccine applications in chickens. Int J Nanomedicine (2020) 15:3303–18. doi: 10.2147/IJN.S241492 PMC7227821 32494131

[51] Wang S Campos J Gallotta M Gong M Crain C Naik E. Intratumoral injection of a CpG oligonucleotide reverts resistance to PD-1 blockade by expanding multifunctional CD8+ T cells. Proc Natl Acad Sci USA (2016) 113(46):E7240–e9. doi: 10.1073/pnas.1608555113 PMC5135381 27799536

[52] Munakata L Tanimoto Y Osa A Meng J Haseda Y Naito Y. Lipid nanoparticles of Type-A CpG D35 suppress tumor growth by changing tumor immune-microenvironment and activate CD8 T cells in mice. J Controlled release Off J Controlled Release Society (2019) 313:106–19. doi: 10.1016/j.jconrel.2019.09.011 31629036

[53] Rothenfusser S Hornung V Ayyoub M Britsch S Towarowski A Krug A. CpG-A and CpG-B oligonucleotides differentially enhance human peptide-specific primary and memory CD8+ T-cell responses in vitro. Blood (2004) 103(6):2162–9. doi: 10.1182/blood-2003-04-1091 14630815

[54] Jie J Liu G Feng J Huo D Wu Y Yuan H. MF59 promoted the combination of cpG ODN1826 and MUC1-MBP vaccine-induced antitumor activity involved in the enhancement of DC maturation by prolonging the local retention time of antigen and down-regulating of IL-6/STAT3. Int J Mol Sci (2022) 23(18):10887. doi: 10.3390/ijms231810887 36142800 PMC9501507

[55] Chen TH Liu WC Chen IC Liu CC Huang MH Jan JT. Recombinant hemagglutinin produced from Chinese Hamster Ovary (CHO) stable cell clones and a PELC/CpG combination adjuvant for H7N9 subunit vaccine development. Vaccine (2019) 37(47):6933–41. doi: 10.1016/j.vaccine.2019.02.040 PMC7115541 31383491

